# Evolving Synergy Between Synthetic and Biotic Elements in Conjugated Polyelectrolyte/Bacteria Composite Improves Charge Transport and Mechanical Properties

**DOI:** 10.1002/advs.202405242

**Published:** 2024-09-11

**Authors:** Samantha R. McCuskey, Glenn Quek, Ricardo Javier Vázquez, Binu Kundukad, Muhammad Hafiz Bin Ismail, Solange E. Astorga, Yan Jiang, Guillermo C. Bazan

**Affiliations:** ^1^ Department of Chemistry and Chemical & Biomolecular Engineering National University of Singapore Singapore 119077 Singapore; ^2^ Singapore Centre on Environmental Life Sciences Engineering (SCELSE) Nanyang Technological University Singapore 637551 Singapore; ^3^ Institute for Functional Intelligent Materials (I‐FIM) National University of Singapore Singapore 117544 Singapore

**Keywords:** abiotic/biotic interface, bioelectrochemical systems, conductive polymer hydrogels, electroactive bacteria, engineered living materials, extracellular polymeric substances, gene expression

## Abstract

gLiving materials can achieve unprecedented function by combining synthetic materials with the wide range of cellular functions. Of interest are situations where the critical properties of individual abiotic and biotic elements improve via their combination. For example, integrating electroactive bacteria into conjugated polyelectrolyte (CPE) hydrogels increases biocurrent production. One observes more efficient electrical charge transport within the CPE matrix in the presence of *Shewanella oneidensis* MR‐1 and more current per cell is extracted, compared to traditional biofilms. Here, the origin of these synergistic effects are examined. Transcriptomics reveals that genes in *S. oneidensis* MR‐1 related to bacteriophages and energy metabolism are upregulated in the composite material. Fluorescent staining and rheological measurements before and after enzymatic treatment identified the importance of extracellular biomaterials in increasing matrix cohesion. The synergy between CPE and *S. oneidensis* MR‐1 thus arises from initially unanticipated changes in matrix composition and bacteria adaption within the synthetic environment.

## Graphical abstract

Combining electroactive *S*. *oneidensis* MR‐1 together with conjugated polyelectrolyte hydrogels increases current output in bioelectrochemical systems. This work investigates the mechanisms of enhancement and shows that these living materials change over time from the initial matrix properties and cellular functions.

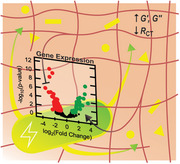

## 1 Introduction

**Figure 1:**
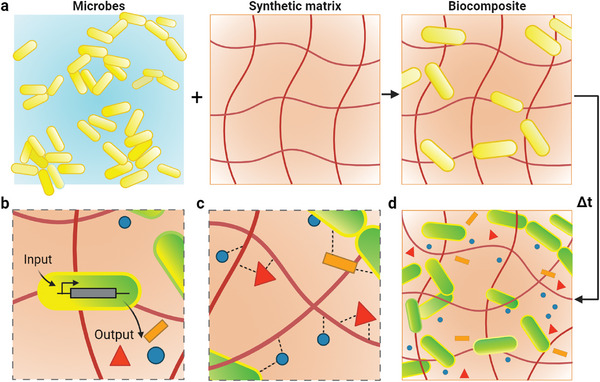
Cartoon schematic of living biocomposite formation and possible synergistic evolution over time. a) Microbes are incorporated with a synthetic matrix to form a biocomposite. b) Microbes encapsulated in the matrix adapt their functions overtime (signified as a color change of the cells from yellow to green) while producing extracellular components. c) Interactions of extracellular components with the cells and synthetic matrix. d) The composition and function of the biocomposite differs from the initial conditions through temporal evolution. Created with BioRender.com.

Living composite materials that incorporate microbial cells within synthetic matrices have attracted interest due to applications in both industry and biomedicine. These applications range from biocatalysis and whole‐cell biosensors to the development of self‐replenishing drug‐eluting materials.^[^ ^1^, ^2^, ^3^ ^]^ In composite materials, the living cells endow the matrix with diverse native and engineered capabilities, including chemical synthesis, sensing, and current generation, while the abiotic component usually functions to confine and stabilize cells across multiple length scales, with tunability of elasticity and porosity.^[^ ^4^, ^5^, ^6^, ^7^ ^]^ Additionally, the abiotic component can contribute features such as stimuli responsiveness, conductivity, and degradability, enhancing the functionality of the composite material. To date, the design and formation of living composite materials typically relies on matching the intrinsic properties characteristic of each component. This alignment is made with the expectation of achieving an additive effect (illustrated schematically in **Figure** **1**a).

Evidence of synergy, wherein the admixture of complementary components beneficially improves the performance of each other, has emerged in the literature.^[^ ^1^, ^7^ ^]^ Microbial cells are well suited in this respect because of their ability to quickly adapt to environmental perturbations, i.e., Figure 1b. For instance, yeast cells immobilized in synthetic hydrogel matrices exhibit slower growth rates, but overall higher metabolism and sustained output leading to increased ethanol and protein production compared to planktonic cells.^[^ ^8^, ^9^, ^10^ ^]^ The enhanced performance is typically attributed to different chemical concentrations and osmotic pressure found in the synthetic matrix compared to those present in the liquid culture.^[^ ^9^, ^11^ ^]^ More recent studies point to the stiffness of the hydrogel network influencing microbial cellular phenotype, but the exact physiological state of encapsulated microbes remains uncertain.^[^ ^12^, ^13^ ^]^ Further, living cells and their extracellular materials can also reinforce synthetic matrices (Figure 1c).^[^ ^14^ ^]^ For example, curli nanofibers produced by engineered *E. coli* established mucoadhesive hydrogels able to persist for extended therapeutic treatments in the gastrointestinal tract.^[^ ^15^, ^16^ ^]^ Meanwhile, immobilized spores of *Bacillus sphaericus* can improve the self‐healing efficiency of concrete and maintain the composite structure by precipitating calcium carbonate.^[^ ^17^, ^18^ ^]^ In all, cellular functions and the composition of the matrix can change over time due to synergistic effects (Figure 1a,d).

**Figure 2:**
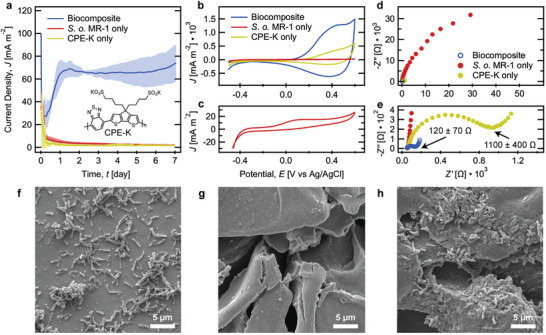
Bioelectrochemical characterization and SEM imaging of biocomposites and controls. a) Chronoamperometry over 7 days, *E*_CA_ = 0.3 V versus Ag/AgCl (*n* = 4). b,c) Cyclic voltammetry on day 7, scan rate = 5 mV s^−1^. d,e) Electrochemical impedance spectroscopy on day 7, *E*_DC_ = 0.3 V versus Ag/AgCl. Annotated values represent the average charge transfer resistance and standard deviation (*n* = 4). Electrochemical cells were supplied with 40 mM lactate in *Shewanella* Basal Media and incubated at 30 °C under anaerobic conditions. Representative SEM images on day 7 of f) the *S. oneidensis* MR‐1‐only biofilm, g) the neat CPE‐K gel, and h) the biocomposite on gold working electrodes.

Bioelectrochemical technologies encompassing the use of electrogenic microbes in biosensors, microbial fuel cells, and bioelectrosynthesis platforms also benefit from the hybridization of living and synthetic components.^[^ ^19^, ^20^, ^21^ ^]^ A particular manifestation is the design of materials for improved electron extraction and injection between microbes and an external electrode.^[^ ^22^, ^23^, ^24^, ^25^, ^26^ ^]^ Recent work toward constructing 3D organizations of cells with conductive materials including reduced graphene oxide, conjugated polymers, and metal oxide nanoparticle/carbon nanotube assemblies has significantly boosted electrochemical performace of devices.^[^ ^27^, ^28^, ^29^, ^30^, ^31^, ^32^ ^]^ A specific example concerns CPE hydrogels, namely CPE‐K (see the inset of **Figure** **2**a), combined with the electrogenic bacterium *Shewanella oneidensis* MR‐1.^[^ ^33^, ^34^, ^35^ ^]^ We have previously shown that composites of *S. oneidensis* MR‐1 with CPE‐K enable biocurrent collection many‐fold larger relative to control biofilms atop gold and carbon electrodes. The improved performance, relative to conventionally used unmodified biofilms, has been attributed to a 3D organization that enables more cells in communication with the electrode and increased current extracted per cell. Further, when compared to the neat CPE‐K hydrogel matrix, the living material shows an order‐of‐magnitude lower charge transfer resistance. These findings support the idea of synergy between the biotic and abiotic components, that is, Figure 1d. However, the extent to which the synthetic matrix affects the function of the microbe and vice versa leading to the enhanced electrochemical properties remains unexplored.

Despite current literature on bacterial metabolism and biofilm development in diverse scenarios, a better understanding is needed of the interactions between microorganisms and their surrounding synthetic matrix in living composite materials. Linking cellular response to engineered microenvironments will allow for more insightful selections of biotic and abiotic components from myriad options and result in a more tailored design of living materials a priori. In this way, desired microbial functions such as sensitivity, selectivity, and turnover rate can be maximized, which would enhance overall current density, power output, and productivity in the case of bioelectrochemical technologies. Herein, we detail how synergy arises in living composites of CPE‐K hydrogels and *S. oneidensis* MR‐1 to provide enhanced bioelectrochemical performance over standard biofilms, using total RNA‐sequencing, fluorescent staining, rheological measurements, and electrochemical testing. These findings connect our understanding of the functions and behaviors of microbes in bioelectrochemical systems to those of encapsulated cells to advance the design of living materials.

## 2 Results

### 2.1 Electrochemical Characterization of Biocomposite, Microbe‐Only Biofilm, and Abiotic Matrix

Living bioelectrochemical composites (biocomposites) were prepared following published protocols.^[^ ^33^ ^]^ Specifically, 1 mL of *S. oneidensis* MR‐1 liquid culture at 0.15 OD was mixed with solid CPE‐K to afford 5 mg mL^−1^ CPE‐K biocomposites. For each replicate, 100 µL of the mixture was deposited atop a gold electrode well via solution casting. Refer to the Supporting Information for experimental details and Figure S1 (Supporting Information) for the electrochemical device design. Note that a higher‐molecular‐weight CPE‐K is used here compared to previous reports (≈32,000 g mol^−1^ vs ≈7,000 g mol^−1^), which allows functional biocomposites to be formed at lower polymer concentrations.^[^ ^36^ ^]^ The optimal CPE‐K concentration for maintaining bacteria viability while still forming a gel was determined by minimum bactericidal concentration testing. The microbes could tolerate [CPE‐K] up to 5 mg mL^−1^ with slightly enhanced microbial growth compared to the control culture (Figure S2, Supporting Information).

The electrochemical performance of the biocomposite was compared to *S. oneidensis* MR‐1‐only, which we will refer to as control biofilms, and neat 5 mg mL^−1^ CPE‐K matrices. Current densities collected over time (chronoamperometry, CA) at 0.3 V versus Ag/AgCl are provided in Figure 2a. Biocomposites reach a steady‐state after two days, generating an average current density ≈30‐fold greater than that of control biofilms after 7 days (74 ± 16 mA m^−2^ vs 2.1 ± 0.5 mA m^−2^, see **Table** **1** for a summary of bioelectrochemical parameters). The CPE‐K‐only matrix produces negligible current. Figure 1f–h shows scanning electron microscopy (SEM) images of electrodes after 7 days. Control biofilms show a sparse monolayer of cells with some microcolonies, while biocomposites show a 3D architecture with cells embedded within polymer aggregate layers. Quantification of live cells at the end of CA reveals that biocomposites support a ≈12‐fold higher cell density than control biofilms. Further, the current extracted per cell, determined by dividing current by cell density, is ≈3‐fold higher in biocomposites (186 ± 36 fA cell^−1^) compared to control biofilms (64 ± 14 fA cell^−1^).

**Table 1:** Bioelectrochemical parameters after 7 days of current collection.

Measured Parameter	Expression	Biocomposite	Control
Current density ^a)^ (mA m^−2^)	J	74 ± 16	2.1 ± 0.5
Cell density^b)^ (× 10^7^)	X	7.8 ± 0.6	0.66 ± 0.21
Current per cell^c)^ (fA cell^−1^)	*I*_cell_ = *JA*/*X*	186 ± 36	64 ± 14
Charge collected (C)	*Q*_col_ = *A*∫*J*(*t*)d*t*	7.7 ± 0.6	0.47 ± 0.11
Lactate consumed^d)^ (mM)	Δ[lac] = │[lac]_f –_ [lac]_i_│	3.4 ± 0.7	1.7 ± 0.7
Lactate charge equivalent^e)^ (C)	*Q*_lac_ = Δ[lac]*VFn*_e_	20 ± 4	10 ± 4
Lactate coulombic efficiency (%)	*CE*_lac_ = 100 × *Q*_col_/*Q*_lac_	38 ± 8	6 ± 2

^a)^ Errors represent standard deviations from the mean (*n* = 4);

^b)^ Calculated from agar plate colony forming unit counting (*n* = 2);

^c)^ Where *A* is the electrode area, *A* = 2 cm^2^;

^d)^ Determined by HPLC (biocomposite *n* = 5, control *n* = 8);

^e)^ Where *V* is the electrochemical cell volume, *V* = 15 mL; *F* is Faraday's constant, *F* = 96485 C mol^–1^; *n*_e_ is the number of electrons available per lactate molecule, *n*_e_ = 4.

Cyclic voltammetry (CV) and electrochemical impedance spectroscopy (EIS) were performed after 7 days of current collection to gain insights into the mechanisms for improved charge extraction from biocomposites (Figure 2b–e). The trends in electrochemical data for the higher‐molecular‐weight CPE biocomposite and abiotic matrix are similar to those reported previously with lower‐molecular‐weight CPE‐K.^[^ ^33^, ^34^ ^]^ Figure 2c shows the CV response of the control biofilm: a redox peak centered near ≈0 V corresponding to direct electron transfer from cells through Mtr membrane proteins to the electrode and an overall sigmoidal shape indicating catalytic current.^[^ ^37^ ^]^ Compared to the control biofilm, the biocomposite and CPE‐K‐only matrix produce higher CV currents. The CV traces are dominated by a quasi‐rectangular response signifying pseudocapacitive behavior, which is more accentuated in the presence of bacteria (Figure 2b).^[^ ^38^ ^]^ The increased area under the curve and lower onset of oxidation in the trace for the biocomposite, compared to the CPE‐K‐only matrix, indicate that the biocomposite material constitutes a matrix with higher electroactive surface area and therefore more continuous interchain contacts for charge transport.^[^ ^36^, ^39^ ^]^ Furthermore, Nyquist plots show a lower charge transfer resistance in the biocomposites than CPE‐K‐only matrices (Figure 2d,e, see the Supporting Information for equivalent circuit fitting). Overall, the increased cell density and improved electrochemical properties of the biocomposite compared to the microbe‐only biofilm and CPE‐K‐only matrix, respectively, reveal biotic/abiotic synergy in the biocomposite to promote enhanced current collection.

### 2.2 Differential Gene Expression of *S. oneidensis* MR‐1 in Response to CPE Matrix Environment

Transcriptomic analysis was employed to investigate changes in gene expression between cells in the control biofilm versus those in the biocomposite matrix. *S. oneidensis* MR‐1 is well suited to this analysis due to its annotated genome and extensive genetic studies into its biofilm formation and respiratory diversity in different environments.^[^ ^40^, ^41^, ^42^ ^]^ Biofilms and biocomposites were harvested from electrodes after 7 days of CA to undergo RNA extraction and purification. Because non‐covalent crosslinks hold together the CPE‐K matrix, dissolution of the biocomposite is facile, however, removal of CPE‐K from the RNA samples was only achieved with additional purification using humic acid removal columns. RNA sequencing and bioinformatics details can be found in the Supporting Information.

**Figure 3:**
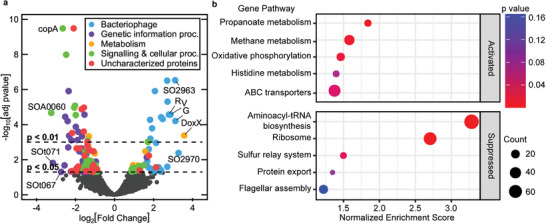
Transcriptomic analysis of *S. oneidensis* MR‐1 in biocomposites versus control biofilms (*n* = 6). a) Volcano plot showing the most significantly differentially expressed genes in color with the top 10 most differentially expressed genes labeled. Fold change refers to gene expression in the biocomposite relative to the biofilm. b) Gene set enrichment plot showing gene sets with the highest absolute normalized enrichment scores that are activated or suppressed in the biocomposite relative to the biofilm. Gene sets are defined by the Kyoto Encyclopedia of Genes and Genomes (KEGG); enrichment score: the degree to which a gene set is overrepresented at the top or bottom of a ranked list of genes, normalization accounts for differences in gene set size; count: the number of genes enriched in each pathway.

Transcriptomic profiles indicate that the expression of a small subset of genes in *S. oneidensis* MR‐1 was affected when comparing cells from CPE‐K biocomposite matrices to those from microbe‐only biofilms, see **Figure** **3**a. A total of 164 genes (3.7% of the total gene content) were significantly differentially expressed between the two conditions (p < 0.5). Most upregulated genes in the biocomposite fell into the functional categories of bacteriophages and metabolism. A large fraction of the most‐highly‐down‐regulated genes in the biocomposite were annotated as genetic information processing and uncharacterized proteins. Several signaling and cellular processing genes were also affected, with upregulation of siderophore‐related genes and the downregulation of type II toxin‐antitoxin systems and cellular motility genes. A complete table of the differentially expressed genes and their relative fold changes (FC) can be found in the Supplemental Information.

#### 2.2.1 Gene Set Enrichment Analysis (GSEA): Energy Metabolism and Electron Transport Chain

GSEA identifies more specific trends within the genes based on known biochemical pathways in *S. oneidensis* MR‐1 (Figure 3b).^[^ ^43^ ^]^ The three gene pathways most significantly activated in the biocomposite over the biofilm concern carbon and energy metabolism: propanoate metabolism, methane metabolism, and oxidative phosphorylation. The activities of these catabolic pathways are important factors in the extracellular electron transfer (EET) activity of current‐generating bacteria.^[^ ^44^, ^45^, ^46^ ^]^ Electrons generated from lactate oxidation in *S. oneidensis* MR‐1 reach the quinol pool in the inner‐membrane via the formate‐dependent (part of methane metabolism) or NADH‐dependent (oxidative phosphorylation) pathways. From there, electrons reach the electrode via Mtr outer‐membrane proteins.^[^ ^42^ ^]^ While the expression of genes in the formate‐dependent pathway (*fdh*, *pta*, *ackA*) are not significantly different between cells in the biocomposite and biofilm, genes related to the higher‐energy‐yielding NADH‐dependent pathway were differentially expressed. NADH‐quinone oxidoreductase genes (*nuo*) showed an average log_2_FC = 1.0 increase while tricarboxylic acid (TCA) cycle enzymes succinyl‐CoA synthetase (*sucCD*, log_2_FC = 1.2, *p* = 0.05) and 2‐methyl citrate dehydratase (*acnD*, log_2_FC = 1.1, *p* = 0.03) also saw upregulation in the biocomposite. Cells in the biocomposite may thus more effectively metabolize lactate to produce more reducing equivalents than cells in the control biofilm. Lactate coulombic efficiency, defined as the fraction of electrons harvested as current relative to the maximum possible electrons available in the lactate consumed,^[^ ^47^ ^]^ was greater in devices containing biocomposites compared to control biofilms (38 ± 8% vs 6 ± 2%, respectively, see Table 1). Previous studies reported that the NADH‐dependent pathway and TCA cycle were induced by increasing the potential of the electron acceptor, that is, the electrode in electrochemical cells.^[^ ^46^, ^48^, ^49^, ^50^ ^]^ This suggests that, relative to control biofilm bacteria, biocomposite bacteria are responding to different extracellular electron acceptor environments provided by the CPE‐K network. Indeed, EIS characterization in Figure 2d,e shows that the CPE‐K‐only matrix and biocomposite exhibit lower impedance than cells atop the bare electrode.

Interestingly, the major components of the EET pathway in the outer membrane (Mtr proteins and cytochromes) critical for electricity generation in MR‐1 did not exhibit differential expression between the conditions. However, expression of riboflavin and FAD synthetase genes decreased in the biocomposite compared to the control – *ribF*, *ribE*, *ribC* decreased an average of log_2_FC = 1.0. This decrease suggests that these secreted electron shuttles are not as critical for mediating electron‐transfer reactions to extracellular electron acceptors, in this case, the CPE‐K polymer matrix. From GSEA in Figure 3b, the flagellar assembly pathway is also suppressed in the biocomposite. Previous reports found that chemotaxis and flagellar assembly protein expression decreased when electron acceptors were in high abundance or at a higher potential.^[^ ^51^, ^52^ ^]^ A decrease in cell motility in the biocomposite is consistent with the CPE‐K matrix being a more readily available acceptor, compared to the bare electrode.

#### 2.2.2 Gene Set Enrichment Analysis: Protein Synthesis

Three pathways suppressed in the biocomposite collectively modulate protein synthesis: aminoacyl‐tRNA synthesis, ribosomal RNA, and the sulfur relay system (Figure 3b).^[^ ^53^ ^]^ Reduced protein synthesis (i.e., slower growth rate) has been associated with nutrient deprivation resulting from high density biofilms or as part of an SOS response to various stressors such as low pH, exposure to magnetic fields, chromate stress, and oxidative stress.^[^ ^54^, ^55^, ^56^, ^57^, ^58^, ^59^ ^]^ The SOS response is an unlikely pretext for protein synthesis reduction in the biocomposite, however, because DNA repair genes *recA* and *lexA* that concurrently activate during SOS conditions were downregulated in the biocomposite (log_2_FC = −1.0, p = 0.03 and log_2_FC = −1.1, p = 0.04, respectively).^[^ ^60^ ^]^ It follows that nutrient deprivation is the more probable cause for the suppression of protein synthesis pathways in the biocomposite. This explanation is consistent with the much higher cell density in the biocomposite compared to the control biofilms at the end of electrochemical testing, see Table 1.

### 2.3 Impact of Biofilm Formation on Biocomposite Properties

One group of genes significantly differentially expressed in the biocomposite encompasses bacteriophages. Their significance is discernable in the volcano plot, but not in GSEA, as bacteriophages do not have a KEGG pathway. Twenty‐seven genes associated with genome‐encoded bacteriophages Lambda (λSo) and Mu (MuSo) showed an average 2.2 log_2_FC (4.5‐fold) increase in expression in the biocomposite. λSo and MuSo phage‐induced lysis promotes biofilm formation and nutrient supply by releasing extracellular polymeric substances (EPS) such as proteins, polysaccharides (PS), lipids, and extracellular DNA (eDNA).^[^ ^61^, ^62^ ^]^ Studies have shown that eDNA in particular is important for cell‐cell and cell‐surface attachment in *S. oneidensis* MR‐1 biofilms.^[^ ^61^ ^]^ Based on the upregulation of genes related to bacteriophages, it occurred to us that the composition of the matrix in the biocomposite would be modified relative to the original CPE‐K‐only content.

**Figure 4:**
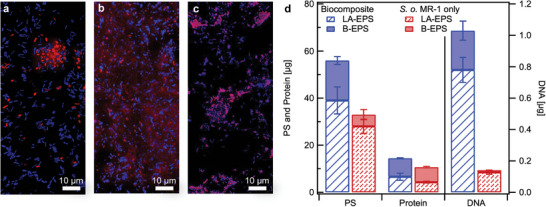
Evaluation of EPS in biocomposites and biofilms. Fluorescent imaging after 7 days of current collection of a) control biofilms and biocomposites b) without and c) with DNAse I treatment. Staining of vital cells was performed using 5 µM SYTO9, whereas staining of dead cells and eDNA was performed with 30 µM propidium iodide. d) Composition of LA‐EPS and B‐EPS in biocomposites and control biofilms (*n* = 3).

The presence of eDNA was first investigated via fluorescent staining. **Figure** **4**a,b shows fluorescent microscope images of the control biofilm and biocomposite atop electrodes after 7 days of current collection. SYTO9 stained all cells (blue) while propidium iodide (PI) was used to visualize dead cells and eDNA (red). The control biofilm appeared to be a thin layer of cells with a few dead cells. In contrast, the biocomposite showed additional fluorescence of the PI as a faint haze outside of the cells with some string‐like structures, indicating the presence of eDNA. After the biocomposite was treated with DNase I to digest single‐ and double‐stranded DNA overnight, there was no signal from PI in the extracellular environment, indicating eDNA had been degraded (Figure 4c). The EPS of control biofilms and biocomposites were extracted to broadly quantify the amounts of DNA, PS, and protein in the extracellular matrix, see the Supporting Information.^[^ ^63^, ^64^ ^]^ Figure 4d summarizes the EPS composition, divided into loosely‐associated (LA) and bound (B) fractions based on the extraction method.

**Figure 5:**
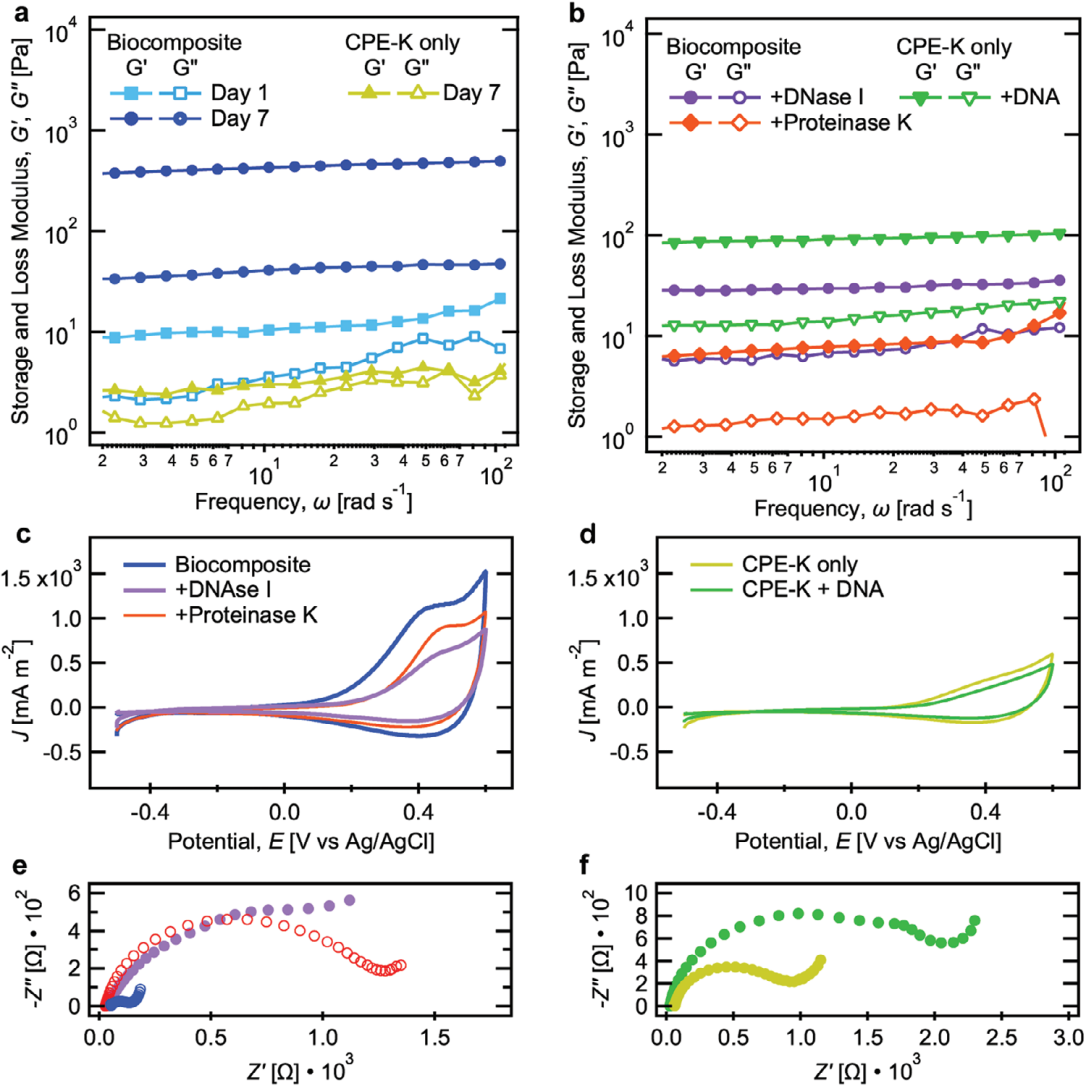
Evaluation of EPS in biocomposite mechanical and electrochemical response. Rheological measurements of the storage (*G*′) and loss moduli (*G*″) as a function of frequency for a) biocomposites and neat CPE‐K gels and b) the aforementioned treated with degradative enzymes or additives, respectively. c,e) CV and EIS of biocomposites with and without DNase I or Proteinase K treatment. d,f) CV and EIS of CPE‐K gels with and without added DNA. Scan rate = 5 mV s^−1^ in CV and *E*_DC_ = 0.3 V versus Ag/AgCl in EIS. Representative traces from *n* = 2.

Electrochemical and rheological responses of biocomposites were measured as a function of time and after enzymatic treatment to determine how EPS modifies relevant properties (**Figure** **5**). In rheological testing, all samples exhibited hydrogel‐like behavior with the storage (*G*′) and loss (*G*″) modulus curves linear and parallel, as well as *G*′ greater than *G*″ across the measured frequency range.^[^ ^65^, ^66^ ^]^ After 12 hours of current collection (day 1) the biocomposite already exhibited higher mechanical strength than the neat CPE‐K gel that had been conditioned for 7 days in the electrochemical cell (average *G*′ = 12 Pa vs 3 Pa), see Figure 5a. On day 7, the mechanical strength of the biocomposite (442 Pa) increased ≈30‐fold compared to day 1. Furthermore, the elasticity of the material also increased (reflected in the decrease in the ratio of *G*″ to *G*′ (tan*δ*) from 0.40 to 0.09).

DNase I and Proteinase K were added to biocomposites to digest single‐ and double‐stranded DNA and proteins, respectively. DNase‐treated biocomposites showed an order‐of‐magnitude decrease in *G*′ compared to the untreated samples (Figure 5b). Treatment with Proteinase K showed a slightly greater effect in disrupting mechanical properties, which could be explained by the larger protein content in biocomposites compared to eDNA (15 ± 2 µg vs 1.3 ± 0.1 µg, from Figure 4d). Enzymatic treatment also decreased the effective electroactive surface area, as seen by the decreased CV area in Figure 5c. Further, charge transfer resistances in treated biocomposites also increased (Figure 5e). Overall, the decrease in desirable electrochemical properties and mechanical strength after enzymatic treatment reveals the significant role eDNA and proteins play in the electrical connectivity and structural formation of the biocomposite.

We examined if adding DNA to neat CPE‐K hydrogels would achieve similar effects to what is observed with endogenous eDNA. DNA (double‐stranded DNA from *E. coli* bacteriophage lambda) was added at 10 ng µL^−1^ (analogous to the concentration found in biocomposites) to 5 mg mL^−1^ CPE‐K and cast atop gold electrodes. After 7 days of conditioning CA, the electrochemical and mechanical properties of the CPE‐K/DNA hydrogels were tested. Adding DNA to CPE‐K increased its mechanical strength and elasticity: 95 Pa, tan*δ* = 0.18 versus 3 Pa, tan*δ* = 0.73 of the neat gel (Figure 5a,b). However, CV characterization showed that DNA/CPE‐K matrices did not achieve the improved electrochemical properties seen in biocomposites (Figure 5d) and charge transfer resistance is adversely affected, showing a doubling in magnitude (Figure 5f). These findings indicate that in situ generation of multiple EPS components by *S. oneidensis* MR‐1 is necessary to concurrently achieve an improvement of mechanical strength and interchain charge transfer in the biocomposite.

## 3 Conclusion

The novelty of the living material described here lies in the spontaneous self‐organization of *S. oneidensis* MR‐1 with CPE‐K to increase biocurrent output in electrochemical devices by virtue of increasing the electroactive area of contact between cells and an external electrode. Indeed, by increasing the dimensionality of electrical contact the current extraction from biocomposites determined by CA increases ≈30‐fold compared to control biofilms atop gold electrodes. Closer inspection of biocomposite properties reveals synergy between the biotic and abiotic components: the current extracted per cell is approximately threefold higher in biocomposites compared to biofilms, while electronic charge transport through the biocomposite is superior to that of the pristine CPE‐K hydrogel matrix, as observed by the lower charge transfer resistance in EIS and larger current density in CV measurements. The latter observation necessitates a more continuous interchain network so that a larger number of CPE‐K polymer chains are electrochemically addressable.

This work surpasses previous performance‐centric studies with CPEs by incorporation of RNA‐sequencing transcriptomics to reveal fundamental changes at a cellular level. *S. oneidensis* MR‐1 in the CPE‐K matrix evolves to direct metabolism toward a more efficient electron transport chain for current generation compared to cells in the microbe‐only biofilm. One also observes upregulation of genes associated with bacteriophages, a feature associated with modulation of biofilm composition. In depth examination of the biocomposite provides new insights regarding the presence of EPS, including PS, eDNA, and proteins. eDNA rigidifies the matrix, as determined by rheological measurements; however, it is an insufficient biological additive for improving the electrochemical characteristic of CPE‐K hydrogels. To what extent the more compositionally complex PS and proteins may combine with eDNA to improve charge transport in the CPE‐K network remains poorly understood. The opportunity exists from a materials design standpoint to tune the synthetic material's interactions with EPS as a route to further current density enhancement. Despite these uncertainties, this work shows that living hybrid materials can be remodeled and optimized through complexation of abiotic components with biomaterials generated *in‐situ* by bacteria. Further insights into how biotic and abiotic synergistic effects can be prearranged a priori create opportunities for designing novel living composites that develop properties beyond those dictated by the initial composition through activation of cellular responses to environmental cues.

## Conflict of Interest

The authors declare no conflict of interest.

## Supporting information

Supporting Information

Supporting Information

## Data Availability

The data that support the findings of this study are available in the supplementary material of this article.
