# Supplementary material for: Evolving Synergy Between Synthetic and Biotic Elements in Conjugated Polyelectrolyte/Bacteria Composite Improves Charge Transport and Mechanical Properties
Source: Adv Sci (Weinh). 2024 Sep 11;11(42):2405242. doi: 10.1002/advs.202405242 (PMC11558123; doi:10.1002/advs.202405242)
Supplement: Supplementary file 1 — Supporting Information [file ADVS-11-2405242-s001.docx]

Supporting Information

**Evolving Synergy between Synthetic and Biotic Elements in Conjugated Polyelectrolyte/Bacteria Composite Improves Charge Transport and Mechanical Properties**

Samantha R. McCuskey, Glenn Quek, Ricardo Javier Vázquez, Binu Kundukad, Muhammad Hafiz Ismail, Solange E. Astorga, Yan Jiang, Guillermo C. Bazan*


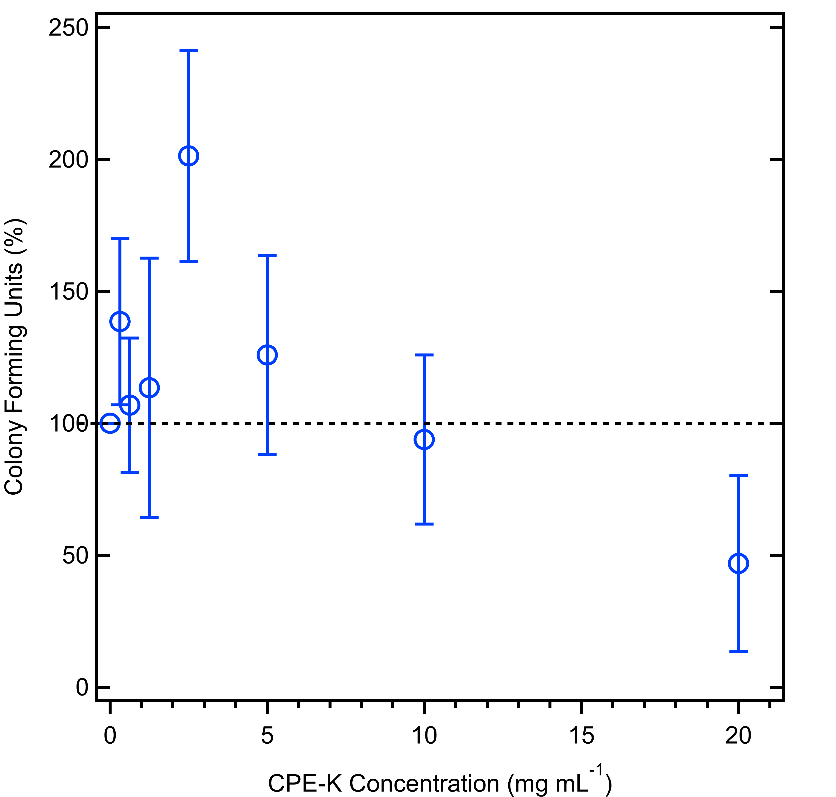


**Figure S1**. Minimum biocidal concentration testing for *S. oneidensis* MR-1 in high molecular weight CPE-K.


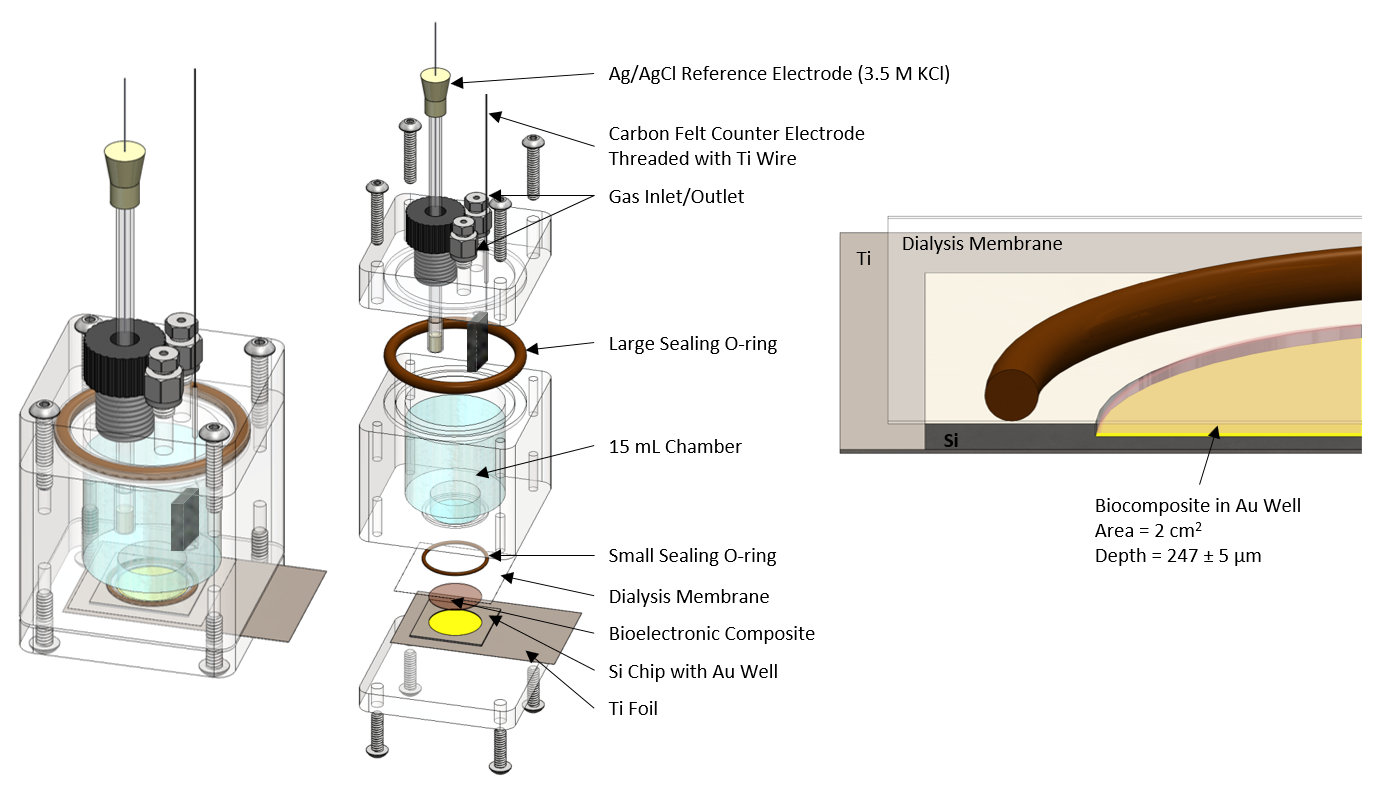


**Figure S2**. Planar-electrode electrochemical cell with dialysis membrane device schematic.

**Experimental Methods**

*Materials*: CPE-K was synthesized according to literature procedures.^[23]^ The molecular weight of CPE-K was determined by GPC of the tetrabutylammonium congener. GPC (DMF): *M*_n_ = 31,959; *M*_w_ = 78,852; *PDI* = 2.47.

*Cell culture and growth medium*: *Shewanella oneidensis* MR-1 (ATCC 700550) was struck out on LB agar plates from frozen stock and incubated at 30 °C to isolate single colonies. Liquid cultures were grown by selecting morphologically similar colonies with a sterile loop to inoculate anaerobic modified *Shewanella* Basal Medium (SBM) containing 20 mM Na-(L)-lactate as electron donor and 20 mM Na-fumarate as electron acceptor. After 16-20 hours incubation at 30 °C with shaking at 120 rpm, cultures consistently reach an OD_600_ of ~0.20 (or 2.0 × 10^8^ cfu mL^-1^). These stationary phase cultures are then centrifuged, washed, and re-suspended in fresh SBM to a final concentration of 0.15 OD_600_.

SBM is a defined media with the following composition per liter: 3 g HEPES buffer, 0.225 g potassium phosphate monobasic, 0.225 g potassium phosphate dibasic, 0.225 g ammonium sulphate, 0.46 g ammonium chloride, 0.117 g magnesium sulphate heptahydrate, 0.5 g casamino acids, 10 mL DL vitamin mix, 10 mL Wolfe’s mineral mix.

*Minimum bactericidal concentration testing*: Various concentrations of CPE-K in SBM with 20 mM Na-(L)-lactate and 40 mM Na-fumarate were inoculated with *S. oneidensis* MR-1 and incubated anaerobically for 18 hours. Because CPE-K absorbs at 600 nm – the standard wavelength at which cell growth is measured in solution – samples were instead diluted and plated on LB agar plates. The number of colonies were counted and the results are summarized in Figure S1.

*Microbial three-electrode electrochemical cells (M3Cs)*: Single-chambered M3Cs were fabricated out of acrylic to have a 15 mL working volume and were sealed with silicone O-rings, see Figure S2 for the device schematic. Working electrode: planar gold well on Si chip, 2 cm^2^, 247 ± 5 μm deep, as fabricated previously.^[20]^ Reference electrode: Ag/AgCl (3.5 M KCl) with 6 mm diameter (Latech, Scientific Supply, Singapore). Counter electrode: carbon felt, ~1 cm^2^, 3.18 mm thick, 99.0% (Alfa Aesar) threaded with 0.25 mm Ti wire (Aldrich). Membrane: regenerated cellulose dialysis membrane, 3.5 kD molecular weight cut-off (Repligen/Spectrum Laboratories). The M3C chamber was kept as a sterile reservoir of 40 mM lactate in SBM. Anaerobic conditions were maintained through constant headspace degassing with humidified, deoxygenated N_2_. Temperature was kept at 30 °C by housing the M3Cs in a temperature regulated incubator.

*Electrochemical characterization*: Chronoamperometry (CA): Using a Bio-logic potentiostat (VSP1, VMP3, or VMP300 models), gold working electrode wells were poised at *E*_CA_ = 0.3 V vs Ag/AgCl to serve as the sole terminal electron acceptor for bacteria. The current response was measured, recorded, and averaged for 30-second blocks with Bio-logic EC-Lab software (EC-Lab V11.36). Cyclic voltammetry (CV): The working electrode potential was swept from *E*_initial_ = –0.5 V to *E*_vertex_ = 0.6 V and back to *E*_final_ = –0.5 V at a scan rate of 0.005 V s^-1^. Electrochemical impedance spectroscopy (EIS): The working electrode potential was poised at *E*_DC_ = 0.3 V vs Ag/AgCl and a sinusoidal potential with amplitude *E*_AC_ = 10 mV vs Ag/AgCl was applied starting from a frequency of 100 kHz to 100 mHz.

EIS spectra were fit to the simplest appropriate equivalent circuit models using Bio-logic EC-Lab software to estimate charge transfer resistance, *R*_CT_. The intersection of the Nyquist curve at the *Z*_Real_ axis in the high-frequency range represents the solution resistance, *R*_S_. The semicircle at high frequency can be modeled with a parallel combination of geometrical capacitance (*Q*_geom_) and charge transfer resistance (*R*_CT_). To fit spectra with a ~45˚ linear response at low frequencies, an additional circuit element called Warburg impedance, *W*, is added in series. This gives the simplest equivalent circuit *R*_S_ + (*Q*_geom_/*R*_CT_) + *W*. For EIS data with a more vertical linear response at low frequencies, an interfacial capacitance (*Q*_int_) is used in place of the Warburg element, giving the equivalent circuit *R*_S_ + (*Q*_geo_/*R*_CT_) + *Q*_int_. In all cases a constant phase element “*Q*” is used in place of a pure capacitor element to represent the deviation from an ideal capacitor.

*Biomass quantification*: Electrodes were removed from the M3Cs and placed in 50 mL centrifuge tubes with SBM. Electrodes were gently agitated with vortexing and pipetting to facilitate dissolution of the biocomposites/biofilms into the media. Suspended cells were then diluted 25 to 1000 more times and plated on LB agar plates. Colonies were counted after incubating the plates at 30 ˚C for 24-48 hours.

*Lactate coulombic efficiency*: An aliquot of sample was removed from the M3Cs on day 7 after the end of electrochemical measurements. High performance liquid chromatography (HPLC) analysis of lactate was performed using an Aminex HPX-87H column (Bio-rad) with the following parameters: 0.008 N H2SO4 mobile phase (0.6 mL min-1), 100 μL injection volume, column temperature of 35 °C and UV detection at 210 nm.

*Scanning electron microscopy (SEM)*: After bioelectrochemical experiments, media in the M3C chambers was exchanged with 4% glutaraldehyde in fresh SBM to chemically fix electrode-associated cells for 24 hours. After fixation, electrodes were sequentially rinsed by exchanging the chamber media with the following solutions twice each for 1.5 hours: SBM media, deionized water, 35% ethanol in deionized water, 70% ethanol in deionized water, 100% ethanol. M3Cs were then disassembled and working electrodes dried via CO_2_ critical point drying. Working electrodes were sputtered with platinum (Anatech Hummer Sputtering System) before imaging with a Hitachi FlexSEM instrument. Accelerating voltage: 5 keV, working distance: ~6 mm, stage current: 50 pA, secondary electron detector.

*RNA extraction, purification, and characterization*: Working electrodes were removed from M3Cs and the biocomposite/biofilm dissolved/scraped with DNA/RNA Shield into sample tubes to preserve cells immediately after electrochemical experiments. Biofilms from multiple control replicates (typically 4) were pooled to yield samples with sufficient RNA for quality control characterization and sequencing. Six replicates were collected for each condition for sequencing (12 samples total). RNA was extracted and purified using the ZymoBIOMICS RNA Mini Kit. For biocomposite samples, CPE-K was removed by Zymo-Spin™ III-HRC Filters. Samples were sequenced at the Singapore Centre for Environmental Life Sciences Engineering (SCELSE) Sequencing Facility on an Illumina Sequencing platform (HiSeq2500 RD, 100bp PE, 2 lanes) using stranded Total RNA library preparation (low input samples). Bioinformatics analysis was performed by Axil Scientific Pte Ltd (Singapore).

*Fluorescence microscopy*: Live/dead staining of cells and eDNA was performed with 5 μM SYTO9, 20 minutes, 30 μM propidium iodide, 10 minutes, with 10 minutes rinsing in between each stain. Samples were viewed using a Leica Thunder fluorescence microscope and 40x objective. Images were processed using LasX software.

*EPS extraction and characterization*: EPS was collected following previously established protocols for *Shewanella* spp. biofilms.^[48, 51]^ To collect loosely-associated EPS (LA-EPS), working electrodes were rinsed with 150 mM NaCl and centrifuged at 4,000 g for 10 minutes, repeated three times. The supernatant was pooled and then filtered through a pre-wet 0.22-micron PES syringe filter. To collect bound EPS (B-EPS), the pellet from the previous step was diluted to 5 mg mL^-1^ wet cell weight basis and mixed in equal parts with 2% EDTA. The mixture was incubated at 4°C for 3 h. The cells were pelleted by centrifugation with 5,000 g at 4°C for 20 min and washed three times using 150 mM NaCl. The supernatant was pooled and then filtered through a pre-wet 0.22-micron PES membrane filter. Polysaccharides were quantified by the phenol-sulfuric acid colorimetric assay using glucose calibration standards. Protein was quantified using the Bradford-Coomassie protein assay with bovine serum albumin (BSA) calibration standards. DNA was quantified using a Qubit dsDNA HS assay.

*Rheology*: Rheological measurements were performed using a Haake Mars 60 (Thermo Scientific) with a plate-cone geometry. The plate diameter and the cone angle were 35mm and 0.5°, respectively, and the gap separation was 0.052 mm. Experiments were performed in a strain-controlled mode at a temperature of 23 °C. 100 µL of sample was deposited onto the plate, and a frequency sweep was done at a constant strain of 0.01%.

*DNA and protein degradation treatment*: DNA was degraded using Promega RQ1 RNase-free DNase I. For the reaction, 1 mM MgSO_4_ was added to electrochemical cell reservoirs and allowed to equilibrate for 30 minutes. The M3Cs were carefully disassembled and DNase I in reaction buffer was added directly to the biocomposite. Untreated samples underwent the same procedure, except reaction buffer-only was added (no DNase I). Protein was degraded using Promega Proteinase K following a similar procedure. Samples were incubated at 30 °C overnight and a second degradation treatment was applied 30 minutes before electrochemical and rheological testing. DNA, lambda (from bacteriophage lambda cl857 Sam, Roche, Sigma-Aldrich) was added at 10 ng µL^-1^ to 5 mg mL^-1^ CPE-K hydrogels.
